# Diversity of Naturalized Hairy Vetch (*Vicia villosa* Roth) Populations in Central Argentina as a Source of Potential Adaptive Traits for Breeding

**DOI:** 10.3389/fpls.2020.00189

**Published:** 2020-02-28

**Authors:** Juan P. Renzi, Guillermo R. Chantre, Petr Smýkal, Alejandro D. Presotto, Luciano Zubiaga, Antonio F. Garayalde, Miguel A. Cantamutto

**Affiliations:** ^1^ EEA H. Ascasubi Instituto Nacional de Tecnología Agropecuaria, Buenos Aires, Argentina; ^2^ Departamento de Agronomía, Universidad Nacional del Sur, Bahía Blanca, Argentina; ^3^ Centro de Recursos Naturales Renovables de la Zona Semiárida (CERZOS), CONICET, Bahía Blanca, Argentina; ^4^ Department of Botany, Palacký University in Olomouc, Olomouc, Czechia; ^5^ Departamento de Matemática, Universidad Nacional del Sur, Bahía Blanca, Argentina

**Keywords:** *Vicia villosa* genotypes, naturalized population, niche-modeling, genetic resource, phenotypic characterization, microsatellites

## Abstract

Hairy vetch (*Vicia villosa* ssp. *villosa* Roth) is native of Europe and Western Asia and it is the second most cultivated vetch worldwide. Hairy vetch is used as forage species in semiarid environments and as a legume cover crop in sub-humid and humid regions. Being an incompletely domesticated species, hairy vetch can form spontaneous populations in a new environment. These populations might contain novel and adaptive traits valuable for breeding. Niche occupancy based on geographic occurrence and environmental data of naturalized populations in central Argentina showed that these populations were distributed mainly on disturbed areas with coarse soil texture and alkaline-type soils. Low rainfall and warm temperatures during pre- and post-seed dispersal explained the potential distribution under sub-humid and semiarid conditions from Pampa and Espinal ecoregions. Conversely, local adaptation along environmental gradients did not drive the divergence among recently established Argentinian (AR) populations. The highest genetic diversity revealed by microsatellite analysis was observed within accessions (72%) while no clear separation was detected between AR and European (EU) genotypes, although naturalized AR populations showed strong differentiation with the wild EU accessions. Common garden experiments were conducted in 2014–16 in order to evaluate populations’ germination, flowering, and biomass traits. European cultivars were characterized by low physical seed dormancy (PY), while naturalized AR accessions showed higher winter biomass production. Detected variation in the quantitative assessment of populations could be useful for selection in breeding for traits that convey favorable functions within specific contexts.

## Introduction

The *Vicia* genus, of the Fabaceae family, includes several winter annual legumes, generically grouped as “vetches.” Within this complex, *Vicia villosa* ssp. *villosa* Roth, commonly known as hairy vetch (HV), is a relevant member. It is native in Europe and West Asia, being introduced as a crop or weed worldwide to temperate climate regions. Hairy vetch is considered a cosmopolitan species due to its high capacity to naturalize under different conditions. It is present in the flora of both South and North Americas, including Argentina (Van de Wouw et al., 2001; Bryant and Hughes, 2011).

Hairy vetch is the second most important vetch in agricultural systems worldwide (Francis et al., 1999). Generally, it is grown for forage, consumed under direct or indirect grazing, or for green manure. In conservation agriculture, the use of HV as cover crop is increasing. Hairy vetch displays high tolerance to biotic and abiotic stresses (Francis et al., 1999). It is one of the recommended cover crop in organic or conservation farming, mainly because it enhances soil nitrogen content by biological fixation (Vanzolini, 2011). Due to its valuable traits, HV could help to improve soil structure, reduce soil erosion, and enhance weed suppression (Clark, 2007; Wayman et al., 2016; Frasier et al., 2017). Typically, HV produces between 2.6 and 6.2 ton ha^−1^ of above-ground dry biomass (Lawson et al., 2015; Mirsky et al., 2017; Ackroyd et al., 2019).

Hairy vetch shows the capacity to form spontaneous populations in ruderal habitats of cultivated areas (Aarssen et al., 1986; Renzi and Cantamutto, 2013). Under natural conditions, the ability to regenerate populations from the soil seed bank is associated with primary combinational seed dormancy (i.e., physical plus physiological dormancy, PY+PD) (Renzi et al., 2014). These naturalized populations can be useful as a genetic resource for breeding. However, despite the high potential agronomic value, HV is an incompletely domesticated species and only a few improved varieties exist. Likewise, HV cover crops are often unreliable in terms of establishment, performance and biomass production (Wilke and Snapp, 2008; Aapresid, 2018). Altogether these drawbacks frequently limit HV adoption by farmers (Maul et al., 2011). Studies concerning geographic distribution and climatic requirements of this species are scarce (Aarssen et al., 1986). The study of the ecological niches of natural HV populations would improve our understanding of its potential adaptation to different environmental factors.

The most important breeding goals of HV include high early vigor, high winter, and spring biomass production and low level of seed dormancy (mainly due to the physical component of primary dormancy). Rapid growth under low temperatures is especially important when HV is used to produce biomass at the end of winter (i.e., cover crop). The time-window for HV growth control (by desiccation or mechanical methods) during early spring, depends on the planting date of the subsequent summer crop. As the growth rate of HV accelerates with the spring advance the adjustment of the control intervention is critical to determine the cover crop performance. Moreover, HV spring biomass production largely determines the amount of nitrogen supplied to subsequent cash crops (Vanzolini, 2011). An additional challenge of HV is seed dormancy control, which can lead to incomplete emergence after seeding (Jacobsen et al., 2010; Maul et al., 2011). On the contrary, in agroecosystems of semiarid regions, HV natural reseeding capacity is a desirable trait reducing establishment costs (Renzi et al., 2017; Renzi et al., 2019), especially when used as forage crop by livestock farmers.

HV was introduced in Argentina more than a century ago (Manganaro, 1919). Since then, several naturalized populations have been established in ruderal habitats surrounding agricultural areas. These populations are considered an unexplored genetic resource for breeding. However, to confirm their potential value as a genetic resource, it is imperative to collect and characterize such material, by comparison, to currently registered cultivated accessions.

The objectives of this study are: i) to describe the natural habitats of naturalized HV populations from Argentina, ii) to assess the phenotypic variability of naturalized populations compared with a set of 41 introduced accessions of HV (including wild and cultivars), and iii) to study the genetic structure using simple sequence repeat (SSR) markers.

## Materials and Methods

### Ecological Characterization

#### Naturalized Populations

**Figure 1:**
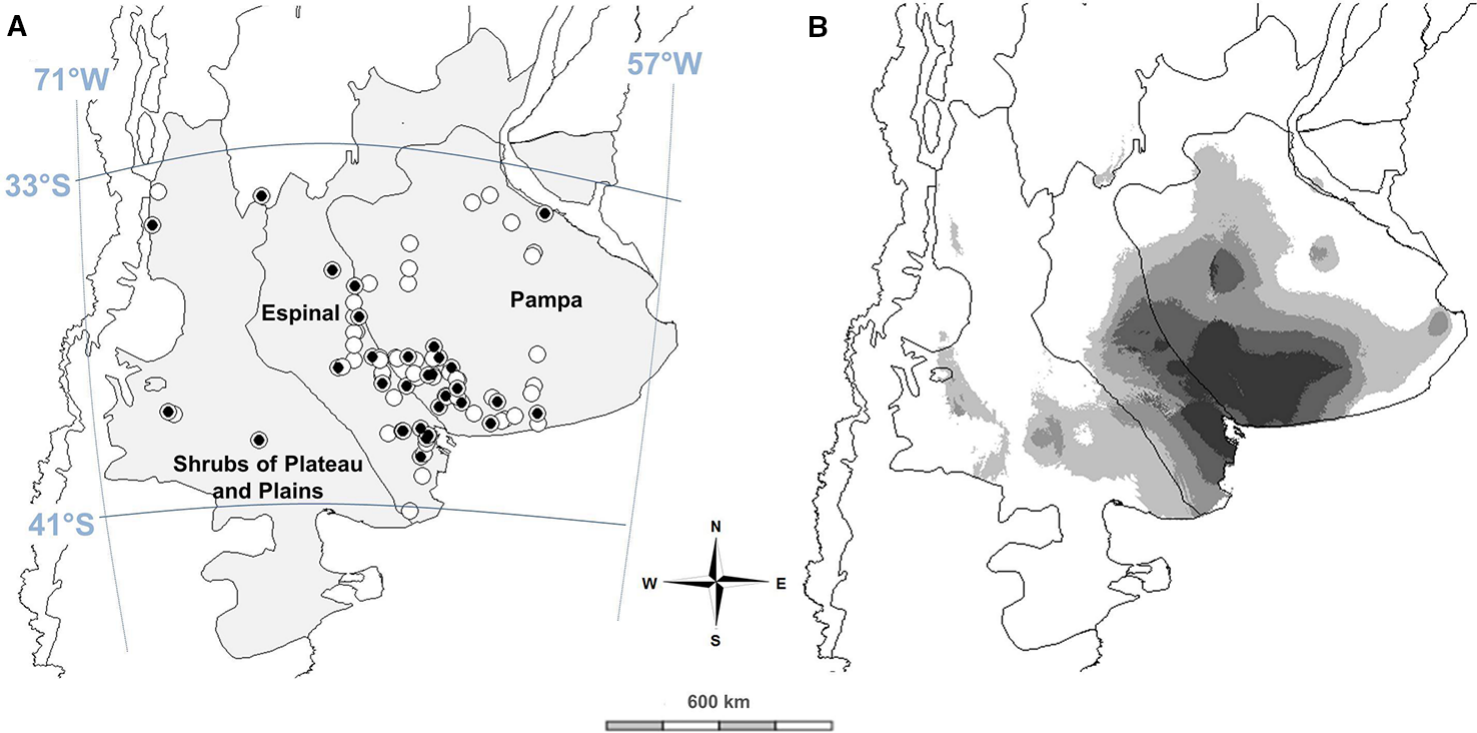
Spatial distribution and seed collection sites (circles) of naturalized hairy vetch populations in Argentina. Gray area shows studied regions and black circles indicate the populations used for phenotypic characterization in the common garden **(A)**. Predicted potential distribution of HV populations in central Argentina based on climatic niche modeling results **(B)**. Lighter colors correspond to lower probabilities of occurrence while darker colors correspond to higher probabilities of occurrence (created with MaxEnt 3.4.1k).

The study area comprised nine provinces: Buenos Aires, La Pampa, Río Negro, Neuquén, Mendoza, Córdoba, San Luis, Santa Fe, and Entre Ríos, belonging to three eco-regions: Pampa, Espinal, and Shrubs of Plateau and Plains (**Figure 1A**). Three exploration trips were accomplished during December 2013-2015, covering a total of 21.400 km. The survey on HV populations was based on specialized systematic bibliography (Burkart, 1952), and voucher specimens deposited at Instituto de Botánica Darwinion (http://www2.darwin.edu.ar) and Museo de La Plata (http://www.museo.fcnym.unlp.edu.ar). To be considered, studied HV populations must be observed for at least two different years at the same locality, and they must contain more than 50 individuals. Recorded information of collection site consisted of i) ecological region, ii) latitude, longitude and altitude, iii) environment (soil and climate) and plant community (dominance of co-occurring plant species) characterized by family, iv) life cycle and origin (Marzocca, 1994). Global positioning system (GPS) coordinates of 63 naturalized populations were recorded (**Table S1**).

#### Environmental Variables

The WorldClim (http://worldclim.org) version 2.0 database was used to extract information about the climate (period 1970–2000). Data were extracted using DIVA-GIS software from ESRI grids with a spatial resolution of 30 arc-seconds (~ 1 km) in the WGS-84 (EPSG: 4326). Bioclimatic variables (BIO1–BIO19) were derived from monthly temperature and rainfall values (Fick and Hijmans, 2017). To avoid over-parameterization, 10 bioclimatic variables were selected to represent annual trends and extreme conditions of temperature and precipitation: annual mean temperature (BIO1), maximum temperature of the warmest month (BIO5), minimum temperature of the coldest month (BIO6), mean temperature of warmest quarter (BIO10), mean temperature of coldest quarter (BIO11), annual precipitation (BIO12), precipitation of the wettest month (BIO13), precipitation of the driest month (BIO14), precipitation of wettest quarter (BIO16), and precipitation of driest quarter (BIO17) (Hijmans et al., 2005). In addition to the bioclimatic variables, edaphic variables related to soil texture, pH, and bulk density were obtained from soil databases (FAO/IIASA/ISSCAS/JRC, 2012) using WGS84 and spatial resolution of 30 arc-seconds. For details, including basic descriptive statistics of each environmental variable see **Table S1**.

Soil samples (at depth of 0–15 cm) were collected at each site to assess the data obtained from soil databases. Soil samples were air-dried and sieved to < 2 mm. pH was measured using a glass electrode pH-meter (soil: water, 1:2.5). Texture analysis (% clay, silt, sand) on HCl and H_2_O_2_ treated and chemically [0.05 M (NaPO_3_)_6_ and 0.15 M Na_2_CO_3_] dispersed samples was carried out by a combination of sieving and pipette methods (Cantamutto et al., 2008; Santos et al., 2017). Pearson’s Correlation Coefficient was performed between soil databases and samples using the InfoStat software (Di Rienzo et al., 2013).

#### Niche Analysis

Ecological niche models were constructed using the geographic locations of HV naturalized populations. Maxent (version 279 3.4.1, Phillips et al., 2018) at default conditions, a maximum-entropy based machine learning method was used for modeling purposes. Maxent showed better performance than other methods when samples sizes are small (Hernandez et al., 2006) and it estimates the potential niche instead of the realized distribution of the modeled entity (Phillips et al., 2006; Hradilová et al., 2019). As environmental predictors, bioclimatic and soil variables at a resolution of 2.5 arc minutes were used. Logistic output with suitability values ranging from 0 (unsuitable habitat) to 1 (optimal habitat) was used. Occurrence points (75%) were used to calibrate the model. The remaining (25%) occurrence points were used for model evaluation. Model strength was quantified using the area under the curve (AUC) of the receiver operator generated within Maxent (Zhu et al., 2017; Phillips et al., 2018).

### Phenotypic Characterization

#### Plant Material

In order to determine the biodiversity of studied naturalized populations (**Figure 1A**, **Table S1**), we compared accessions originating from Argentina (AR) and Europe (EU). Twenty-nine naturalized populations were evaluated in a common garden (2014 and/or 2016) experiment (**Table 1**). They were selected based on the amount of available collected seed stock (> 30 g) and wide distribution (**Figure 1A**). Cultivated germplasm from AR consisted of 10 varieties (landraces) maintained by farmers (**Table 1**) and two registered cultivars (Tolse F.C.A and Ascasubi INTA) (www.inase.gov.ar).

**Table 1:** Country, improvement status (cultivar, wild, and naturalized), and name of hairy vetch accessions included in the phenotypic and genotypic studies.

Country of origin	N	Accession/locality^†^ (name)	Evaluation**
Cultivar			
Hungary	1	Kartali	a
	2	Simabuekkoeny	a
Poland	3	Rea	a
	4	Rod MPI	a
	5	Sielecka	a,b,c
Turkey	6	617 81	a,b
	7	Capello	a,b
Bulgaria	8	266 99	a,b,c
Czech Republic	9	Arida	a
	10	HS 1884	a
	11	Modra	a,b
	12	nsl. Dobrenice	a
	13	Troubsko	a,b,c
	14	Viola	a
France	15	Savane	a,b
Germany	16	Ebsdorfer	a
	17	Oregon	a,b
	18	Polyp	a
	19	SAM 21	a
	20	Welta	a
Russia	21	Pridesnjanskaja	a
	22	Stavcanka	a,b,c
	23	Stenskaja 24	a
Serbia	24	Sarajevo	a
Wild			
Czech Republic	1	Pouzdrany^†^	a
	2	Zavojno jezero I^†^	a
Serbia	3	Petrovo Selo^†^	a,b
	4	Radenka II ^†^	a,b,c
	5	Senokos^†^	a
Naturalized			
Argentina	1	Algarrobo^†^	a,b
	2	Bordenave^†^	a
	3	C. Dorrego^†^	b
	4	C. Pringles^†^	b
	5	C. Suarez^†^	a
	6	Chelforó^†^	b
	7	Doblas^†^	a
	8	E. Martini^†^	b
	9	E. S. Pablo^†^	a
	10	G. Acha^†^	b
	11	Guaminí^†^	a,b
	12	Guatraché	b
	13	I. Rico^†^	b
	14	Médanos^†^	a
	15	Ombucta^†^	a
	16	P. Carretas^†^	b
	17	P. Luro^†^	b
	18	Pasman^†^	a
	19	Pigue^†^	a,b
	20	Rancul^†^	a
	21	Rivera^†^	b
	22	S. Luis^†^	b
	23	S. Pedro^†^	a,b,c
	24	Saldungaray^†^	a,b,c
	25	T. Arroyos^†^	b
	26	T. Origone^†^	a
	27	T. Picos^†^	a,b
	28	Tratayen^†^	b
	29	Winifreda^†^	a
Cultivar*			
Argentina	1	Algarrobo^†^	a
	2	Ascasubi INTA	a,b,c
	3	Bordenave^†^	a
	4	Buratovich^†^	a
	5	Carhué^†^	a
	6	G. Chavez^†^	a
	7	Guatraché^†^	a
	8	M. Juárez^†^	a
	9	Oriente^†^	a
	10	Pergamino^†^	a
	11	T. Origone^†^	a
	12	Tolse F.C.A	a,b,c

*Include VNS, variety not stated.

**Phenotypic common garden evaluation in 2014 (a) and 2016 (b), and genotypic analysis with SSR (c).

^†^Correspond to name of a locality.

Wild (n = 5) and cultivated germplasm (n = 24) of HV from EU was represented by 29 accessions (**Table 1**). Origin and accession name of each cultivar were provided by the Research Institute of Crop Production (CRI) of the Czech Republic (**Table 1**; for more information see https://grinczech.vurv.cz/gringlobal/search.aspx) (Renzi et al., 2016).

#### Common Garden

Plants (**Table 1**) were cultivated at the Experimental Agricultural Station (EEA) of Hilario Ascasubi (Buenos Aires, Argentina; 62°37′W, 39°23′S) during 2014 and 2016 growing seasons. The predominant climate in this location is semiarid-temperate with 489 mm mean annual precipitation and 14.8°C mean annual temperature (EEA H. Ascasubi, 1966–2018). The soil was an entic haplustoll, sandy loam, slightly alkaline (pH ≈ 7.5), high in phosphorus (P) content (≈ 22 ppm P Bray & Kurtz) and low organic matter content (≈ 1.6%) at 20 cm (Renzi et al., 2016). Weather data from each year were registered at the nearby meteorological station (less than 500 m) (http://inta.gob.ar/documentos/informes-meteorologicos).

The accessions were arranged in row plots in a randomized complete block design, with three replications. Each experimental unit consisted of a row of 2.50 meters sown with 30 seeds on 10^th^ April 2014 and 27^th^ April 2016. Original collected seeds were used in 2014 and 2016 experiments. Seeds were inoculated with commercial inoculum (*Rhizobium leguminosarum* bv *viciae*) immediately before sowing (Deaker et al., 2004).

To determine the number of days from sowing to 50% flowering, the growing stage was recorded twice a week. After 50% flowering, leaf length (mm), leaflets per leaf, and the number of flowers per raceme were measured on 10 randomly selected individual stems in each plot. For foliar observations, the leaf of the third upper node of the stem and the basal leaflet of the leaf were chosen. Above-ground total dry matter was measured at end of winter (mid-September) and late spring (mid-December) by cutting plant shoots at ground level in a 0.50 m row in each plot. The biomass of each accession, in each year, was expressed in relation to the average biomass per year. Maturity was defined by the presence of 75% ripe pods, approximately at the beginning of summer. Seeds from mature pods were immediately harvested and threshed by hand, on 21^st^ December 2014 and 26^th^ December 2016, for physical (PY) testing. Moisture content at harvest was ≤ 14% (Renzi and Cantamutto, 2013).

#### Statistical Analysis

Analyses of variance (ANOVA) considering a randomized complete block, between improvement status (EU Cultivar, Wild, AR Cultivar, Naturalized; **Table 1**) and between accessions for each improvement status, were performed using InfoStat software. Accessions and improvement status means were compared by Fisher’s least significant difference test. Correlations between quantitative traits were calculated using Pearson’s correlation coefficient. Canonical variate analysis (CVA) was performed with all phenotypic traits based on Euclidean distance through InfoStat software.

#### Physical Dormancy Testing

After the harvest from the common garden experiment, seeds were cleaned and seed weight was estimated, in a sample of 100 seeds in 2014 (n = 1) or 50 seeds in 2016 (n = 3). PY seeds (i.e., “hard” or impermeable) were determined by an imbibition test performed at 20 ± 2°C for 3 days (Baskin and Baskin, 2014). Intact non-germinated seeds of each replication were placed on moist ﬁlter paper in Petri dishes and watered daily with tap water. Imbibed seeds showed a visible change in its size/volume ratio, and were easily distinguished from non-imbibed ones (Renzi et al., 2014). Seed viability of non-germinated seeds was assessed by slicing longitudinally with razor and immersion in a 0.5% (wt/vol) tetrazolium chloride (2,3,5-triphenyltetrazolium chloride) (Sigma-Aldrich) solution for 24 h at 30°C in the dark (ISTA, 2019). Seeds with pink or red-stained embryos were considered viable. The total number of viable seeds consisted of germinated plus stained one (Renzi et al., 2016).

For all accessions, PY break dynamics as a function of storage time (38 days) and temperature (20°C) under wet conditions were analyzed using the area under the curve (AUC) calculated by GraphPad Prism Software (GraphPad, San Diego, California, USA). Where AUC = 1 indicates seed without PY (Initial non-PY seeds = 100%) and AUC = 0 indicate PY seed (final non-PY seeds = 0%) (Renzi et al., 2016). Accessions were grouped by improvement status and further compared by Fisher’s least significant difference test using InfoStat software.

#### Relationships Among Phenotypic Traits, Geography, and Environment

To assess relationships between phenotypic traits in 16 and 20 naturalized populations of Argentina (2014 and 2016) and both geographic and environmental distances, six matrices were prepared and examined using the Mantel test (Smouse et al., 1986). The physical distance between naturalized populations was estimated using geographic distance (GGD) for latitude (x)/longitude (y) values: $\mathrm{GGD}=\sqrt{{\left(\mathrm{xi}-\mathrm{xj}\right)}^{2}+{\left(\mathrm{yi}-\mathrm{yj}\right)}^{2}}$ (Peakall and Smouse, 2006; Garayalde et al., 2011). The geographic matrix contained pairwise geographical distances while phenotypic distance was calculated as Euclidean distances between populations. All environmental variables were standardized and were calculated using soil and climatic variables (“environmental”). The significance of the normalized Mantel coefficient was calculated using a two-tailed Monte Carlo permutation test with 1,000 permutations using InfoStat software.

### Genotypic Characterization

#### Plant Sampling and DNA Isolation

Three representative naturalized populations (n = 3) were contrasted with AR cultivar (n = 2), EU cultivar (n=4), and wild accession (n = 1) (**Table 1**). Seeds were sown in a common garden at the Experimental Agricultural Station (EEA) of Hilario Ascasubi. Leaf material from 10 randomly selected plants from each accession was collected at the vegetative stage (August 2016). Genomic DNA was extracted using a modified cetrimonium bromide (CTAB) method (Hoisington et al., 1994) from leaf tissue dried on silica gel.

#### Microsatellite Markers

The five most polymorphic SSR markers were chosen (**Table S2**) from set of 36 simple sequence repeat (SSR) markers developed by Raveendar et al. (2015) for common vetch (*Vicia sativa* subsp. *sativa*) (Chung et al., 2013) and being applicable for HV genotyping analysis. Amplification reactions were performed in 17 μl volumes containing: 0.25 U of Taq DNA polymerase (Invitrogen), 1 mM MgCl2, 1.1 pmol of primers, 1 mM of each deoxynucleoside triphosphate (dNTP), and 30 ng of genomic DNA template. The optimum annealing temperature was determined for each primer set: KF008505 (55°C), KF008507 (59°C), KF008512 (59°C), KF008526 (59°C), and KF008536 (60°C). Amplifications were initially checked on 1.5% agarose gels. PCR products were analyzed on 6% denaturing polyacrylamide gel, 1×TBE electrophoresis buffer at 60 W for 75 min and the bands were visualized by silver staining and scanned (modified from Tang et al., 2003 and Garayalde et al., 2011). The size of each SSR allele was estimated using a 100 bp molecular weight marker. Each DNA fragment was considered as an allele of a single co-dominant locus.

#### Genetic Data Scoring and Genetic Variability

The amplified SSR loci were scored for 10 accessions. Homozygous and heterozygous genotypes were inferred from the band patterns and allele frequencies (*pi*) calculated accordingly. The absence of band (null allele) was scored as missing data. Mean expected heterozygosity values (*He*) and the percentage of polymorphic loci (*P%*) were calculated: $HE=1-\sum pi^{2};\;P\%=\frac{Lp}{LT}\times 100$, where *pi* is the frequency of the *i*th allele, *Lp* is the number of polymorphic loci, and *LT* is the total number of loci. Hardy–Weinberg equilibrium was tested using chi-squared test $X^{2}=x^{2}=\sum_{i=1}^{k}\frac{{\left(Oi-Ei\right)}^{2}}{Ei}$, where *O_i_* is the observed number of individuals of the *i*th genotype, *E_i_* is the expected number under equilibrium hypothesis, and *K* is the total number of genotypes. Degrees of freedom for the chi-squared test were calculated as d.f. = [Na(Na–1)]/2, where Na is the number of alleles at the locus (following Garayalde et al., 2011).

The calculation of genetic distances (GD) followed the method of Peakall et al. (1995) and Smouse and Peakall (1999). For the analysis of a SSR single-locus, the first step involves the calculation of the vector by additive genotype scoring convention per individuals. Subsequently, the squared distance (*d^2^*) between any two genotypes is one-half the Euclidean distance between their respective pair of vectors as follows: $d_{ij}^{2}=\frac{1}{2}\overset{k}{\underset{k=1}{\sum }}{\left(y_{\mathrm{ik}}-y_{\mathrm{jk}}\right)}^{2}$, where *i* and *j* are the genotypes and *k* is the scoring character. Squared distances range from 0, when individuals share the same alleles, to 4 when individuals are homozygous for different alleles. Genetic distance matrices for each locus were summed across loci under the assumption of independence. At population level, a Ø*_PT_* (analogue of *F*_ST_) obtained from analysis of molecular variance (AMOVA) was used as an estimate of population genetic differentiation with SSR markers. Principal coordinate analyses (PCO) were performed on GD matrices. The correlation between genetic distance and phenotypic matrix was analyzed by the Mantel test (see Garayalde et al., 2011).

#### Analysis of Molecular Variance

The individual pairwise GD matrices were subjected to AMOVA. Total genetic variation was partitioned into three levels: within and between accessions and between origin (AR and EU). Variation was summarized both as the proportion of the total variance and as *Ø*-statistics (Excoffier et al., 1992). Genetic variability measures, distance metrics, PCO analysis, correlation analysis, and AMOVA were analyzed using GenAlEx 6 (Peakall and Smouse, 2006).

## Results

### Ecological Niche of Naturalized Populations

Naturalized populations of hairy vetch were found in the three monitored regions (**Figure 1A**), corresponding to Pampa, Espinal, and Shrubs of Plateau and Plains. In the central temperate region of Argentina, annual rainfall varies from semi-arid to arid conditions with only 200 mm, to sub-humid and humid environments with approximately 1,000 mm. The vegetation changes in all three regions, from arid steppes in the west (Shrubs of Plateau and Plains) to grass steppes without woody species in the east (Pampa). The Espinal is an intermediate savannah, with grasses and scarce xeric trees, mainly of the *Prosopis* genus.

The proposed niche modeling explained most of the variation in HV geographical distribution. The area under the receiver operating curve (AUC) score of MaxEnt models, both training and test AUC values, were 0.957 and 0.956, respectively, indicating that most climatically suitable areas predicted by MaxEnt were highly correlated with the occurrence of natural HV populations. The distribution was significantly affected by precipitation amount of the driest quarter (BIO17), max temperature of the warmest month (BIO5), annual mean temperature (BIO1), and clay content in the soil surface (t_clay, **Table 2**). The main suitable habitats for HV are distributed in the southeast of Espinal and southwest of the Pampa region, characterized by sub-humid and semiarid temperate climates, with warm-dry summers and cold-wet winters (**Figure 1B**).

**Table 2:** Contribution (%) of the bioclimatic and soil variables in the MaxEnt models, and suitable habitats of naturalized hairy vetch populations from Argentina.

Environmental variables	Contribution	Suitable habitats	Unit
	(%)	Mean (range)	
Precipitation of driest quarter	27.2	25.5 (16.6–39.3)	mm month^−1^
Max temperature of warmest month	16.2	25.9 (24.1–27.7)	°C month^−1^
Annual mean temperature	15.2	12.3 (11.0–13.6)	°C month^−1^
Clay content in the surface soil	11.5	28.3 (8.0–38.0)	%
Annual precipitation	5.0	632 (4,007–812)	mm year^−1^
Soil pH	4.7	6.6 (6.1–9.7)	
Sand content in the surface soil	4.6	37.1 (23.0–84.0)	%
Min temperature of coldest month	4.3	1.5 (0.3–2.5)	°C month^−1^
Mean temperature of coldest quarter	3.6	6.6 (5.7–7.3)	°C month^−1^
Precipitation of driest month	2.9	20.5 (9.0–27.0)	mm month^−1^
Silt content in the surface soil	2.6	34.6 (8.0–49.0)	%
Mean temperature of warmest quarter	0.9	18.3 (16.8–19.8)	°C month^−1^
Precipitation of wettest quarter	0.8	71.7 (44.6–91.7)	mm month^−1^
Precipitation of wettest month	0.4	84.4 (51.0–103.0)	mm month^−1^
Soil bulk density	0.1	1.3 (1.2–1.6)	kg dm^−3^

Plant communities associated with HV comprised of 63 species (**Table S1**). Most frequent species were cosmopolitan weeds, including *Avena fatua*, *Cynodon dactylon, Sorghum halepense* (Poaceae), *Carduus* sp., and *Centaurea solstitialis* (Asteraceae) and natural communities of the perennial pasture *Festuca arundinacea* (Poaceae). **Table 3** presents the life cycles and origins of the 20 species frequently associated with naturalized HV populations in the explored region, considered as the dominant community species. Exotic species represent 85% of the co-occurring vegetation.

**Table 3:** Dominant community species associated with hairy vetch populations in central Argentina.

Species	Family	Cycle	Origin	Frequency
*Avena fatua* L.	Poaceae	A	E	0.50
*Carduus nutans* L. and *C. acanthoides* L.	Asteraceae	A	E	0.45
*Cynodon dactylon* (L.) Pers.	Poaceae	P	E	0.42
*Centaurea solstitialis* L.	Asteraceae	A	E	0.40
*Sorghum halepense* (L.) Pers.	Poaceae	P	E	0.38
*Festuca arundinacea* Schreb.	Poaceae	P	E	0.38
*Stipa ambigua* Speg. *and A. caudate* Trin.	Asteraceae	P	Na	0.37
*Taraxacum campylodes* G.E. Haglund	Asteraceae	P	E	0.33
*Diplotaxis tenuifolia* (L.) DC.	Brassicaceae	P	E	0.29
*Melilotus albus* Medik.	Fabaceae	A	E	0.28
*Medicago lupulina* L.	Fabaceae	A-P	E	0.28
*Ammi majus* L.	Umbelliferae	A	E	0.24
*Lolium multiflorum* Lam.	Poaceae	A	E	0.22
*Dactylis glomerata* L.	Poaceae	P	E	0.22
*Rapistrum rugosum* (L.) All.	Brassicaceae	A	E	0.22
*Eragrostis curvula* (Schrad.) Nees	Poaceae	P	E	0.20
*Erigeron bonariensis* L.	Asteraceae	A	Na	0.20
*Plantago lanceolata* L.	Plantaginaceae	P	E	0.19
*Medicago sativa* L.	Fabaceae	P	E	0.15
*Bromus catharticus* Vahl	Poaceae	B	Na	0.14

Life cycle: A, annual; P, perennial; B, biannual. Origin: Na, native; E, exotic.

### Plant Growth and Phenotypic Variability

Registered rainfall in EEA Hilario Ascasubi during HV growing season (from April to December) was 50% higher in 2014 (498 mm) and 21% lower (264 mm) in 2016, compared to historical long-term means (331 mm). Mean daily air temperature values were slightly higher in 2014 (13.5°C) compared to 2016 (12.8°C) growing season. All AR accessions performed well, except for Tolse F.C.A in 2014. However, nine out of the twenty-four EU tested cultivars (nr. 1, 7, 9, 10, 12, 13, 15, 16 and 19; **Table 1**) and two out of the five wild populations (1 and 2) did not produce pods during 2014.

**Figure 2:**
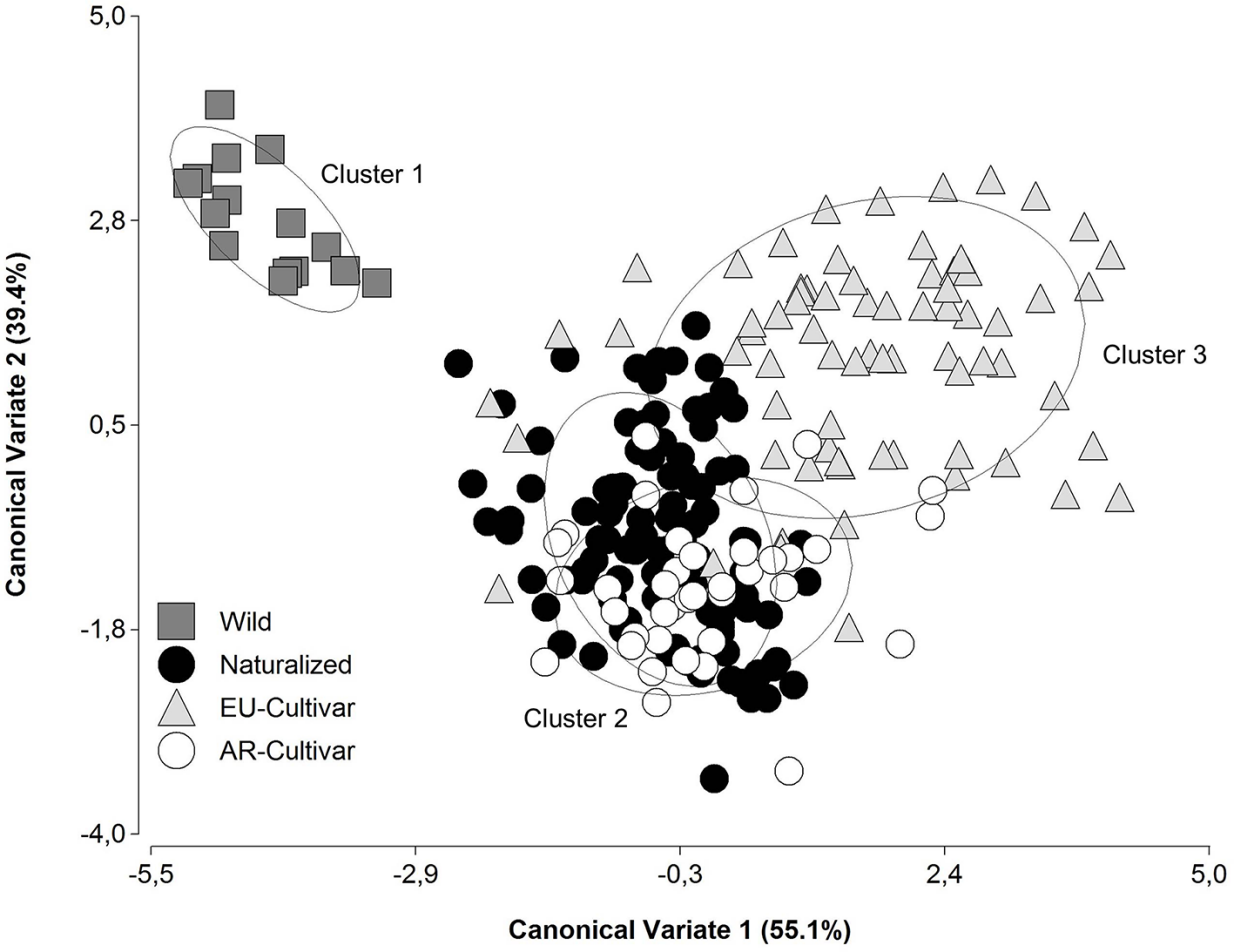
Scatterplot of the four improvement status groupings on the two canonical discriminant functions based on phenotypic traits in a common garden during 2014 and 2016.

Using canonical discriminant analysis with the phenotypic traits, the accessions were grouped in three clusters (**Figure 2**). These corresponded to improvement status and origin. Cluster 1 consisted predominantly of wild populations, cluster 2 consisted accessions of AR origin, and in cluster 3 were EU cultivars.

AR accessions showed higher winter biomass production compared to EU cultivars and wild genotypes (**Table 4**). Biomass in spring was less variable between improvement status. Over the total data set, HV winter biomass was negatively correlated to the number of leaflets per leaf (r = -0.20; P < 0.01), and the spring biomass was positively correlated with the leaf length (r = 0.74; P < 0.001). There was strong positive correlation between winter and spring biomass (r = 0.40; P < 0.001). Variations in the days to flowering among improvement status were narrow (≤ 7 days) but statistically significant within status (**Table 4**).

**Table 4:** Phenotypic variability of hairy vetch (HV) for each improvement status and origin (means and range) grown in a common garden during 2014 and 2016 in Experimental Agricultural Station (EEA) Hilario Ascasubi.

Improvement status	Origin	n	Relative biomass^†^		Leaf length	Leaflet number	Flower number	Days to		Physical dormancy	Seed	
			Winter	Spring				Flowering	Maturity		Viability	Weight
					(mm)	(n)				AUC	(%)	(mg)
Cultivar	EU	24										
Mean			0.69^B^	0.94^B^	57	15.3^B^	26^B^	195^AB^	245^AB^	0.72^D^	97^A^	33^B^
Range			(0.10–1.84)	(0.35–1.59)	(35–83)	(12–19)	(10–40)	(145–203)	(204–255)	(0.15–0.99)	(84–100)	(21–42)
Within status (p < 0.05)			**	**	**	*	**	**	**	**	**	**
Cultivar	ARG	12										
Mean			1.47^D^	0.99^BC^	59	14.7^A^	23^A^	197^BC^	248^B^	0.48^C^	98^AB^	32^B^
Range			(0.72–2.39)	(0.37–1.63)	(22–81)	(10–19)	(13–28)	(169–202)	(221–255)	(0.11–0.88)	(88–100)	(27–41)
Within status (p < 0.05)			NS	**	**	*	NS	**	**	**	*	**
Naturalized	ARG	30										
Mean			1.27^C^	1.06^C^	58	15.2^B^	25^B^	193^A^	241^A^	0.39^B^	99^B^	31^B^
Range			(0.26–2.35)	(0.48–1.89)	(32–99)	(13–19)	(18–32)	(178–202)	(221–260)	(0.08–0.66)	(88–100)	(22–40)
Within status (p < 0.05)			**	**	**	**	NS	**	**	**	*	**
Wild	EU	5										
Mean			0.20^A^	0.77^A^	51	17.2^C^	21^A^	200^C^	246^AB^	0.13^A^	96^A^	15^A^
Range			(0.01–0.59)	(0.23–1.07)	(37–77)	(15–20)	(15–33)	(188–213)	(233–255)	(0.00–0.23)	(76–100)	(10–21)
Within status (p < 0.05)			*	**	*	**	NS	**	NS	NS	**	*
Between status (p < 0.05)			**	**	NS	**	**	*	**	**	**	**
LSD			0.15	0.08		0.5	2	4	4	0.06	1	2
Status * Year			NS	NS		**	NS	NS	**	NS	NS	NS

For mean values, different letters indicate significant differences among status (Fisher’s LSD test, α = 0.05).

‘**’Indicates significance at p < 0.01 and ‘*’ at p < 0.05.

^†^Relative to mean biomass per year (winter 222 and 168 g m^−1^ and spring 1,174 and 887 g m^−1^ in 2014 and 2016, respectively).

NS, not significant.

Seed viability was over 80% in all cases. The area under the curve (AUC), showing the following dormancy gradient rank: EU cultivars < AR cultivars < naturalized < wild genotype. No significant interaction between improvement status x year was found in the AUC (**Table 4**). Wild genotypes had a smaller seed weight (**Table 4**).

**Figure 3:**
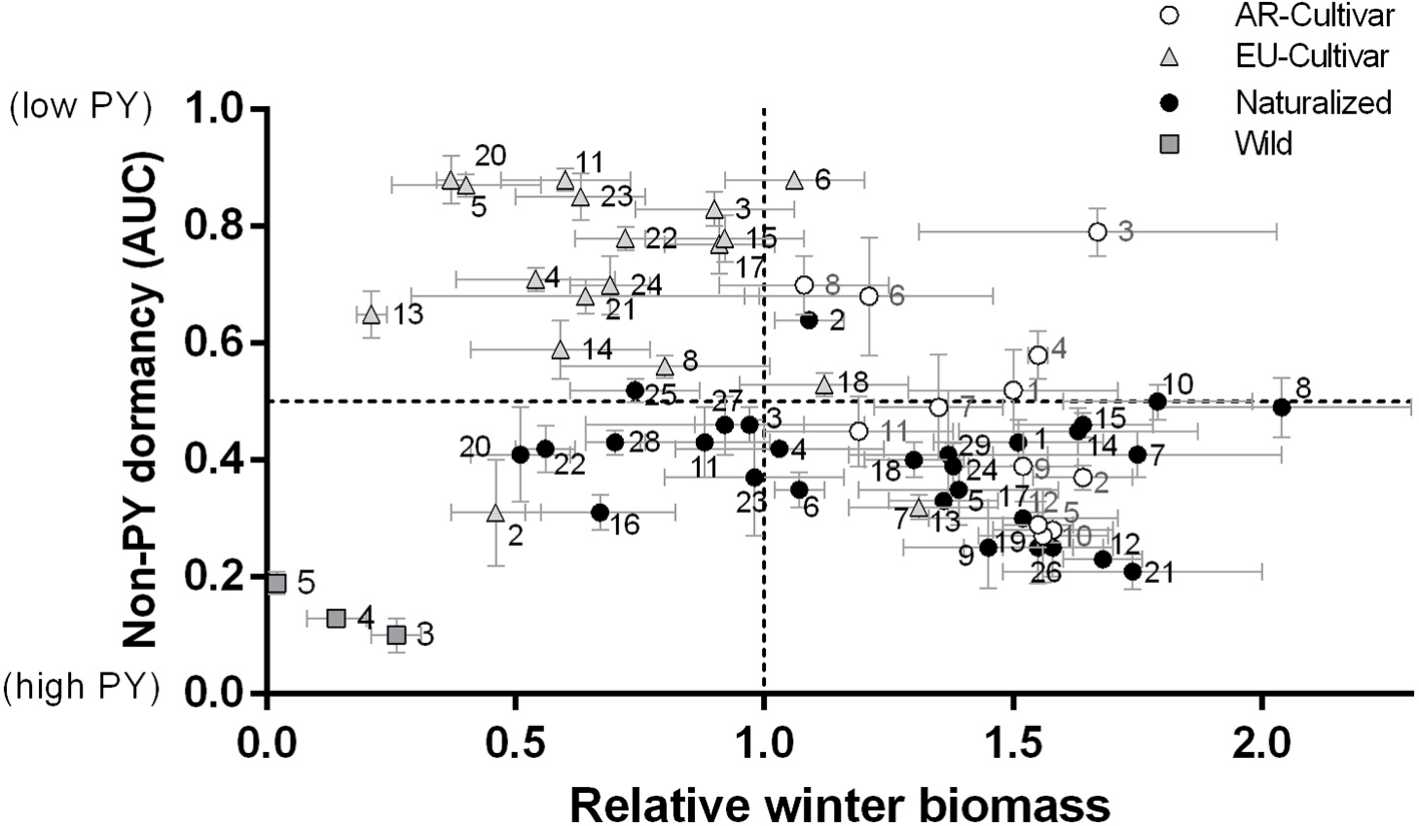
Relationship between relative winter biomass and physical (PY) dormancy [area under the curve (AUC)] for each genotype (mean and standard error) evaluated in common garden during 2014 and 2016. For number references see **Table 1**.

**Figure 3** shows the relationship between the main evaluated traits (winter biomass and seed dormancy), in order to identify the more suitable genotypes for breeding programs. Accessions number 9, 12, 19, 21, 26 of naturalized populations and 2, 5, 10, 12 of AR cultivars showed better potential for winter biomass with high PY. While the genotypes number 3, 6, 8 for AR cultivars, 2 for naturalized populations and 6 for EU cultivar showed better potential for both winter biomass and low PY.

### Genotypic Characterization

The five selected polymorphic SSR loci produced altogether 24 alleles in 100 tested individuals. The mean number of alleles per locus (N) was 4.8 ± 0.9 ranging from 3 to 8 among the five loci. The mean expected heterozygosity (He) was 0.667 ± 0.03.

**Figure 4:**
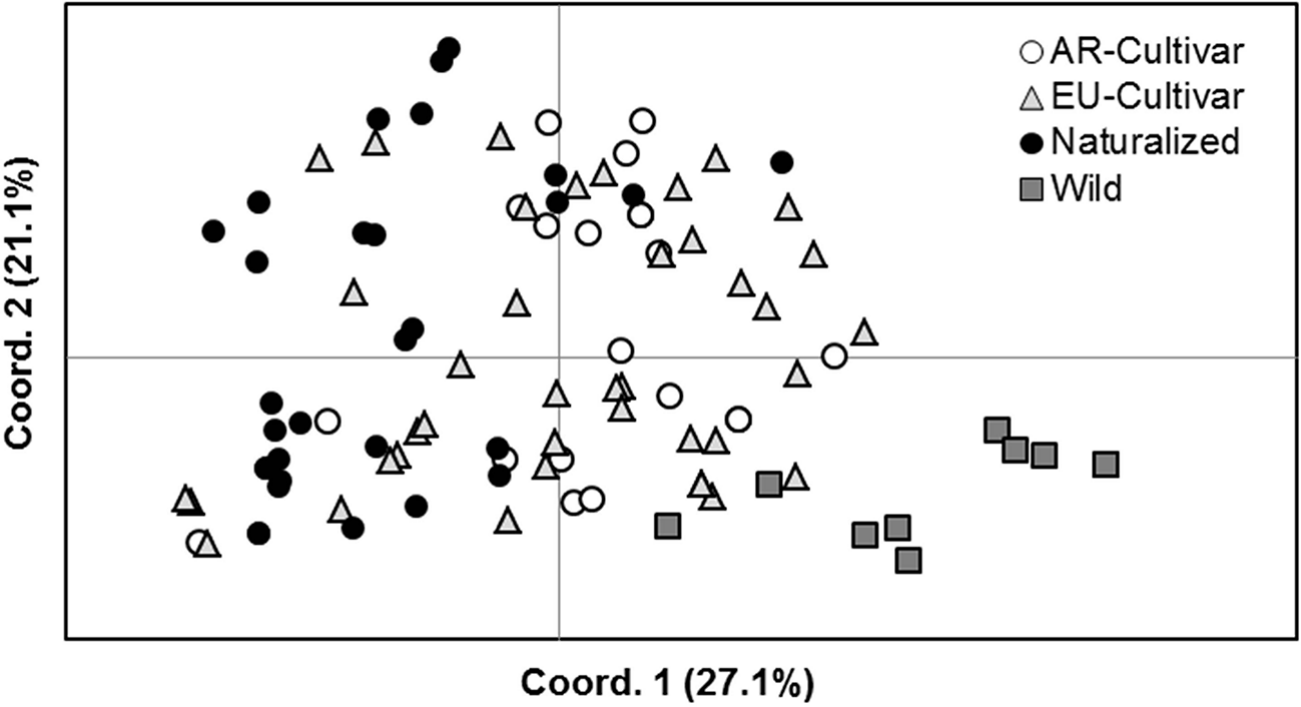
Principal coordinate analyses (PCO) plots based on the individual simple sequence repeat (SSR) distance matrix.

Differences in variability were observed between improvement status, being higher in EU cultivars (He = 0.64 ± 0.03; N = 4.4 ± 1.0) and naturalized populations (He = 0.63 ± 0.05; N = 4.6 ± 0.9) compared to AR cultivars (He = 0.57 ± 0.06; N = 3.8 ± 0.9) and wild (He = 0.30 ± 0.09; N = 2.2 ± 0.4) genotypes. The lower value of variability in wild ones might be due to the smaller number of analyzed individuals (n = 10). Two private alleles were found only in naturalized populations. Equilibrium tests were significant in the 68% of cases, indicating non-random mating within populations (**Table S3**). AMOVA showed a significant differentiation between improvement status, which explained around 19.1% of the variance. Naturalized populations differed from the wild accession (a genetic differentiation of 46.7%) and also from EU (8.3%) and AR (14.3%) cultivars. Lower differentiation was found between AR and EU cultivars (4.3%) (**Figure 4**).

AMOVA for the total marker data set is shown in **Table 5**. Genetic diversity was high within accessions. Between-accession estimated variance was significant and around 25% of the total variation. A small but significant portion of variance (3%) was found attributable to differences between AR and EU genotypes.

**Table 5:** Analysis of molecular variance (AMOVA) and sources of variation for hairy vetch accessions.

Source of variation	df	SS*	MS**	% variation	*p-value*
Among origin	1	24,097	24,097	3	<0,02
Among accessions	8	136,098	17,012	25	<0,01
Within accession	90	338,600	3,762	72	<0,01
Total	99	498,795		100	

*SS, sum of squares.

**MS, mean sum of squares.

### Relationships Among Distance Measures

Environmental distance matrices were significantly correlated with geographic distance matrices (Mantel test; environment: r_2014_ = 0.61, P < 0.01; r_2016_ = 0.78, P < 0.01), suggesting that environmental (climatic + soil) conditions diverge with increasing geographic distance. However, geographic (r_2014_ = 0.19, P = 0.16; r_2016_ = 0.10, P = 0.20) nor environmental (r_2014_ = -0.04, P = 0.54; r_2016_ = 0.15, P = 0.10) distances were not significantly correlated with phenotypic distance.

Genetic and phenotypic distance matrices of naturalized populations showed a positive but non-significant statistical correlation (Mantel test; r = 0.19, P = 0.21).

## Discussion

### Ecological Niche

Argentinian hairy vetch populations occur on a transitional zone between two defined ecological regions, Pampas with sub-humid climate and Espinal with semiarid conditions (**Figure 1**). HV was used as a forage species in Buenos Aires province before 1900 (Manganaro, 1919). Thereafter, it probably escaped from cultivation (Aarssen et al., 1986) and became naturalized. As HV natural seed dispersal potential is very limited (Jannink et al., 1997), seed spillage due to handling and transportation was probably the most important distribution method. Human-mediated dispersal is the most likely explanation of establishment and subsequent naturalization of HV into further suitable habitats (Horvitz et al., 2017; Pascher et al., 2017).

After its introduction in Argentina, HV spread over the areas which met the appropriate conditions, following a patchy distribution. HV showed adaptation in broad geographic (33–41°S, latitude, 60–66°W longitude) and climatic range (400–800 mm rainfall; 11–13.6°C annual mean temperature; **Table 2**). Duke (1981) mentioned that HV is well adapted to a greater range of annual mean temperatures between 4.3–21°C. In this study, low rainfall and warm temperatures during summer months explained HV potential distribution of natural populations (**Table 2**). HV was generally associated with neutral-alkaline (range 6.1–9.7 pH), sandy or sandy loam soils. However, it could occur on most soil types with sufficient drainage capacity (Duke, 1981). Clark (2007) stated that HV preferred neutral (pH 6.0–7.0) soils with tolerance to alkalinity (Duke, 1981). Conversely, low pH (< 6.2) can decrease the rate growth, nodulation, and nitrogen fixation (Aarssen et al., 1986).

### Fitness for the Ecological Niche

The ability of HV to produce PY+PD dormant seeds and their subsequent germination and emergence are important factors that influence natural population dynamics and persistence (Kimball et al., 2010). During the period of seed formation, dry and warm conditions could shorten the species life cycle due to rapid thermal-time accumulation (Petraityte et al., 2007) as well as favor a decrease on seed moisture content. In HV, the acquisition of PY is initiated only when the moisture content of the seeds is ≤14% (Hyde, 1954). Furthermore, HV is a cross-pollinated species where bees play an important role (Zhang and Mosjidis, 1995; Renzi et al., 2017), thus dry and warm weather is favorable for the activity of pollinating insects (Petraityte et al., 2007; Al-Ghzawi et al., 2009).

In the humid central region of Argentina, the spread of naturalized populations of HV would be limited by a negative combination of two main factors. First, the abundant rainfall stimulates the virulence of foliar fungal diseases (e.g., *Ramularia sphaeroidea* Sacc. and *Ascochyta viciae* Lib.), which reduce photosynthetic leaf area thus limiting seed formation (Petraityte et al., 2007; Renzi and Cantamutto, 2013). In addition, high humidity conditions enhance HV indeterminate growth, non-uniform maturity and extended growing season favoring a biennial behavior (Duke, 1981). These consequently limit the seed formation, drying, and acquisition of PY, and can cause a sharp reduction of seed bank persistence and consequent natural regeneration, similar to observations of Toser and Ooi (2014) in *Acacia saligna*.

After the seed dispersal, warm summer temperatures are required for seed dormancy release (Renzi et al., 2014; Renzi et al., 2016). Seed dormancy acquisition, release, and seedling emergence requirements are important fitness traits determining ecological niches of HV (**Figure 1B**). These adaptive traits seem to have evolved in Mediterranean-like environments, where hot and dry summer conditions regulate seed dormancy alleviation while cool and wet winters contribute to enhance vegetative growth providing safe-sites for seedling recruitment (Van Assche and Vandelook, 2010; Picciau et al., 2019).

### Phenotypic Variability

Two cultivars of woolly-pod vetch (Capello and Tolse F.C.A) were found among AR and EU cultivars. This subspecies (*V. v.* ssp *varia*) is characterized by shorter leaves (29.8 ± 6 *vs*. 47.8 ± 9.3 mm in HV, P < 0.01), fewer leaflets (12.3 ± 1.9 *vs*. 15.2 ± 1.1, P < 0.01); and flowers (20.1 ± 7.7 *vs*. 26.9 ± 3.4, P < 0.01), early flowering and maturity (more than 2 week before, P < 0.01), higher winter (**Figure 3**) but lower spring biomass in relation to HV (≈ 60%, P < 0.01). On the other hand, Jannink et al. (1997) found that accessions of HV were more winter-hardy than woolly-pod vetch, and late flowering may be positively genetically correlated with winter hardiness (Maul et al., 2011). The susceptibility of HV to low-temperature increases with more advanced phenological stages (Brandsaeter et al., 2002) while slow growth during winter can be an adaptation attribute to avoid frost damage (Loi et al., 1993). Prompt maturity is a desirable trait for earlier biomass production as well as N accumulation in regions with a shorter growing season. Also, flowering timing can greatly influence the capacity for weed-suppression as a cover crop (Mischler et al., 2010; Maul et al., 2011). Therefore, woolly-pod vetch could be a source of desirable genes for breeding program seeking early flowering cultivars for cover crop usage in mild winter zones. Notably, most of the genotypes characterized as early flowering by Maul et al. (2011) corresponded to woolly-pod vetch (https://npgsweb.ars-grin.gov/gringlobal/search.aspx).

Quantitative traits evaluated among accessions resembled a continuous probability distribution, although the difference between improvement status was statistically significant (**Table 4**). AR accessions showed higher winter biomass accumulation compared to EU, probably due to a greater adaptation to Argentinian ecological conditions.

Seed dormancy is largely genetically determined but also depends on the environmental conditions experienced by the mother plant (maternal effect) and the subsequent degree of seed dehydration (Hudson et al., 2015; Finch-Savage and Footitt, 2017). HV seeds were collected from mature pods with seed moisture content less than 14% (determined as a critical value for PY acquisition, Hyde, 1954), and the environmental effect between years was not detected on AUC of PY (**Table 4**). PY was variable among genotypes and could act mainly as an adaptive trait (Hudson et al., 2015; Long et al., 2015). EU cultivars had lower PY values compared to AR cultivars and naturalized accessions. PY is a highly heritable trait (Hudson et al., 2015) thus it could be useful germplasm for both breeding and artificial (or natural) selection for higher or lower levels of dormancy (Lacerda et al., 2007). It is probable that observed differences between accessions could be explained by genetic adaptations of HV to the local environment (Baskin and Baskin, 2014) as shown in pea (Hradilová et al., 2019) or by selection for improved genotypes (Fuller and Allaby, 2009; Kluyver et al., 2013; Renzi et al., 2016).

Observed large variability within available germplasm could be used by breeders to select parental accessions for HV improvement breeding program that maximizes the winter-spring biomass with low PY for cover crops (Wilke and Snapp, 2008; Wayman et al., 2016), or with high PY for ´ley farming´ systems (Loi et al., 2005; Renzi et al., 2017; Renzi et al., 2018). No significant correspondence was found between the geographic distance matrix and the phenotypic distance matrix (P > 0.15) among the naturalized AR populations and these results differ from *Medicago polymorpha* L., in which a correspondence between collection site and phenotypic traits (Loi et al., 1993; Helliwell et al., 2018) was observed.

Among the measured traits there was a significant correlation between winter and spring biomass. The latter was also highly correlated with the length of the leaf as described in *Vicia sativa* (De La Rosa et al., 2002). Leaf size is related to the photosynthetic rates which in turn affect growth and could be potentially maximized with water availability (Carlson et al., 2016). Thus, leaf size would be an indirect selection trait for biomass that improves breeding program efficiency.

### Genotypic Variability

There is scarce information on genetic diversity of HV and therefore the knowledge of genetic variability is useful for other studies. Our data showed low genetic differentiation between AR and EU cultivars, but strong differentiation between wild EU and naturalized AR populations. Similarly to other outcrossing species, variation between accessions was small in comparison with the variance found within populations (**Table 5**), which is expected for an obligate cross-pollinated species (Hamrick and Godt, 1989). Maul et al. (2011) reported similar results, with 93% of genetic diversity within populations.

The Hardy-Weinberg equilibrium was not stable within populations (**Table S3**) and this can be attributed to mutations, natural selection, non-random mating, genetic drift, and gene flow. As mentioned above, a lack of geographical signature in the pattern of population variation, as occurs with other allogamous species (Garayalde et al., 2011), can also be explained by human activities on seed dispersal and genetic drift (Knapp and Rice, 1998). Cultivated populations are open-pollinated and highly heterogeneous and would be subject to natural selection and genetic drift throughout the cycles (Wiering et al., 2018). These results are consistent with observed in the phenotypic traits.

## Conclusions

This study increases the understanding of the value of naturalized hairy vetch populations in agroecosystems of Argentina. Naturalized populations showed good soil adaptation in disturbed areas and neutral response to alkaline soil niches from central Argentina. Low rainfall and warm temperatures during pre- and post-dispersal seem to explain and regulate the potential distribution of HV populations. Within this ecological context, dry and warm climate may be considered as favorable environmental conditions to increase seed dormancy and timing of germination-triggering. Considering HV genetic variability and agro-ecological adaptation, naturalized populations could be considered as a source of potential adaptive traits for breeding. The AR germplasm constitutes an important reservoir of genes for high winter and spring biomass production. On the other hand, high levels of innate seed dormancy of HV accessions from Argentina reduce its possible use as a cover crop. In this sense, dedicated crosses with more domesticated EU cultivars will serve to reduce the seed dormancy.

## Data Availability Statement

All datasets generated for this study are included in the article/**Supplementary Material**.

## Author Contributions

JR and MC conceived the topic. JR and LZ performed the experiments. JR and AG analyzed all statistical data. JR, GC, and AP wrote the manuscript. PS and MC revised the manuscript.

## Funding

This work was supported by the Instituto Nacional de Tecnología Agropecuaria (PE-E6-I146). Agencia Nacional de Promoción Científica y Tecnológica. MINCyT (PICT-2017-0473 and PICT-2016-1575) and Universidad Nacional del Sur (PGI 24/A223 and PGI 24/A225).

## Conflict of Interest

The authors declare that the research was conducted in the absence of any commercial or financial relationships that could be construed as a potential conflict of interest.
